# Whole-Genome Sequence Comparisons of *Listeria monocytogenes* Isolated from Meat and Fish Reveal High Inter- and Intra-Sample Diversity

**DOI:** 10.3390/microorganisms10112120

**Published:** 2022-10-26

**Authors:** Larissa Murr, Ingrid Huber, Melanie Pavlovic, Patrick Guertler, Ute Messelhaeusser, Manuela Weiss, Matthias Ehrmann, Christian Tuschak, Hans Bauer, Mareike Wenning, Ulrich Busch, Nancy Bretschneider

**Affiliations:** 1 Bavarian Health and Food Safety Authority (LGL), 85764 Oberschleissheim, Germany; 2 TUM School of Life Sciences, Technical University of Munich, 85354 Freising, Germany; 3 Bavarian Health and Food Safety Authority (LGL), 91058 Erlangen, Germany

**Keywords:** cgMLST, pangenome, SNP calling, genetic diversity, processing plants, outbreak, fish, ST382

## Abstract

Interpretation of whole-genome sequencing (WGS) data for foodborne outbreak investigations is complex, as the genetic diversity within processing plants and transmission events need to be considered. In this study, we analyzed 92 food-associated *Listeria monocytogenes* isolates by WGS-based methods. We aimed to examine the genetic diversity within meat and fish production chains and to assess the applicability of suggested thresholds for clustering of potentially related isolates. Therefore, meat-associated isolates originating from the same samples or processing plants as well as fish-associated isolates were analyzed as distinct sets. In silico serogrouping, multilocus sequence typing (MLST), core genome MLST (cgMLST), and pangenome analysis were combined with screenings for prophages and genetic traits. Isolates of the same subtypes (cgMLST types (CTs) or MLST sequence types (STs)) were additionally compared by SNP calling. This revealed the occurrence of more than one CT within all three investigated plants and within two samples. Analysis of the fish set resulted in predominant assignment of isolates from pangasius catfish and salmon to ST2 and ST121, respectively, potentially indicating persistence within the respective production chains. The approach not only allowed the detection of distinct subtypes but also the determination of differences between closely related isolates, which need to be considered when interpreting WGS data for surveillance.

## 1. Introduction

*Listeria monocytogenes* (Lm) is a pathogenic bacterium capable of the adaption to different environmental niches. Consumption of food contaminated with this pathogen poses a risk to certain groups of people, as it can cause severe infections with complications such as meningitis and encephalitis if the host is immunocompromised (elderly, neonates, and the immunosuppressed) [1].

Due to its high case fatality rates (ranging between 13.0% and 17.6% in the European Union (EU) between 2018 and 2020 [2], for instance), listeriosis is a major issue for food safety and public health institutions worldwide. The epidemiologic surveillance involves the collection and interpretation of epidemiological data, ideally supported by the identification and analysis of potential outbreak-related isolates. The latter can be referred to as clonal or phylogenetically related, exhibiting common pheno- or genotypic traits that can be distinguished from those of epidemiologically unrelated isolates [3]. Consequently, phylogenetic methods based on the comparison of whole genomes provide the highest resolution for microbial typing [4,5].

Frequently used approaches for whole-genome sequencing (WGS)-based outbreak investigations rely on the identification of single-nucleotide polymorphisms (SNPs) or allele differences in gene-by-gene comparisons [5,6]. A popular example for the latter is the detection of allelic differences (ADs) within a set of hundreds or thousands of core genome genes, known as core genome multilocus sequence typing (cgMLST). Here, the sequences determined for a defined set of core genome loci are usually compared with alleles of the respective loci already deposited in a corresponding database in order to assign allele numbers. The resultant allelic profile can be summarized to a so-called cgMLST or complex type (CT). In recent years, the entirety of distinct genes determined for a set of genomes, designated as the pangenome, emerged as a valuable base for additional genome comparisons [6,7,8]. Pangenome analyses enable the comparison of the genetic constitution between different isolates [6,9].

In addition to such high-resolution approaches, WGS data can be used for the determination of subtypes previously derived via subtyping methods with less discriminatory power, such as serotyping or multilocus sequence typing (MLST). For Lm, subtyping schemes are available for serogrouping [10], MLST [11], and cgMLST [12,13,14], which allow the derivation of the respective subtype from a genome sequence. The MLST scheme of Ragon et al. (2008) [11] is based on the allele calling of seven housekeeping genes and allows the classification of isolates into sequence types (STs), which can be combined in clonal complexes (CCs) when differing by a maximum of one allele from at least one other profile of a CC. For Lm, cgMLST usually maintains backward compatibility with MLST [12,13,14]. As the isolates of some MLST CCs are suspected to be hypervirulent [15], in silico MLST can be applied for a preliminary assessment of the infectious potential. Moreover, screening for specific virulence or persistence determinants enables an in-depth characterization of the analyzed isolates.

WGS-based methods have contributed to the identification of outbreak sources in several listeriosis outbreaks [16]. Nevertheless, the interpretation of WGS-based analyses presents a critical factor. The validity of these analyses can strongly depend on the number of included isolates originating from a potentially outbreak-related processing plant. The occurrence of more than one subtype within processing plants was reported previously [17,18,19,20,21,22]. Analysis of a small number of isolates can lead to a failure in detecting different subtypes present within a given environment, and thus, matches between clinical isolates and the infection source may be missed [23].

Furthermore, strict distance thresholds cannot be defined for clustering of potential outbreak-related isolates. Distances need to be assessed in consideration of the variety of genomic diversity within different bacterial populations as well as bacterial transmission within the food production chain and between food and humans [24]. The genetic diversity between isolates from outbreak-related food samples might be slightly lower than the genomic diversity between these isolates and the respective clinical isolates, as isolates may evolve during the course of disease [25]. Therefore, it is important to analyze not only the occurrence of distinct subtypes but also the genetic diversity between isolates of the same subtype in order to assess the applied clustering thresholds. Examinations of the genetic diversity of spatially linked isolates are often based on classifications into subtypes, such as CCs or CTs [23,26]. In outbreak investigations, a close relatedness between isolates should be confirmed by additional methods enabling an in-depth analysis of the genetic diversity of closely related isolates [27].

In this study, we investigated the genetic diversity of 92 Lm meat- and fish-associated isolates by WGS-based typing and characterization. Isolates were classified via serogrouping, MLST, cgMLST, as well as screening for genetic traits and prophages. In order to determine the genetic diversity of (potentially) related isolates, more detailed analyses were performed: besides the ADs detected with cgMLST, differences in accessory genome regions were examined by SNP calling and pangenome analysis.

This approach was applied on genome sequences of: (i) an internal control in order to examine the reproducibility of the described workflow, (ii) meat- and fish-associated isolates obtained from the same samples or processing plants to determine the genomic diversity of spatially linked isolates and to assess the suggested clustering thresholds, and (iii) fish-associated isolates from various origin to investigate potential bacterial persistence or transmission based on the determined genetic diversity. Overall, the proposed combination of methods allows the identification of distinct subtypes and, moreover, case-specific sources of genomic variance between potentially closely related isolates. This information can be used to thoroughly assess the relatedness of isolates, e.g., in outbreak investigations.

## 2. Materials and Methods

### 2.1. Isolate Collection

Ninety-two Lm isolates derived from eighty-one samples from food and food-production plants collected within the official monitoring of foodstuffs in Bavaria, Germany, were analyzed. Of these isolates, 54 were obtained from either the same meat samples or from samples belonging to the same meat-processing plants (set A to E). The residual 38 isolates were originated from fish and fish products (set F). The isolates were divided and analyzed as distinct sets to investigate the genetic diversity of isolates sharing a common feature, i.e., being isolated from the same sample, the same plant, or the same matrix (fish). The samples combined within each set had no known link to samples of one of the other sets. Information about the different sets concerning common features, isolation source, year of sampling, as well as the number and IDs of the respective isolates is provided in Table 1. As an internal control, an additional isolate (LMO-C) was carried along through the entire workflow from cultivation to sequencing for 16 different sequencing runs.

### 2.2. Genus Identification

All isolates were stored at −80 °C using the MAST Cryobank system (MAST Diagnostica GmbH, Reinfeld, Germany) and cultivated on tryptone soya blood agar (Oxoid Deutschland GmbH, Wesel, Germany) at 37 °C for 24 h. The genus was determined by matrix-assisted laser desorption ionization-time of flight mass spectrometry (MALDI-TOF MS) using the MALDI Biotyper (MBT) platform (Bruker Daltonics, Billerica, MA, USA) with the commercial databases MBT Compass Library_RUO_7854 (BDAL 8.0.0.1), SR database, filamentous fungi v1.0 (Bruker Daltonics), as well as an inhouse database. Classification was performed via Biotyper 3.1 software. Sample preparation for MALDI TOF MS analysis was performed according to “the on target extraction protocol” described by Pavlovic et al. (2013) [28].

### 2.3. DNA Extraction

For DNA extraction, the PureLink Genomic DNA Mini Kit (Thermo Fisher Scientific, Waltham, MA, USA) was used according to the manufacturer’s protocol for Gram-positive bacteria with the following modifications for optimization of the DNA purity: The incubation steps at 37 °C and 55 °C were extended from 30 min to 45 min each. Before incubation at 55 °C, 20 μL of RNase A (supplied with the kit) were added and mixed by brief vortexing. The volume of ethanol (≥99%, Carl Roth GmbH + Co. KG, Karlsruhe, Germany) was raised from 200 μL to 350 μL, and the whole lysate volume was transferred to a spin column. An additional centrifugation step (20,000× *g*, 3 min) was implemented after discarding the filtrate of the second washing step to remove residual wash buffer. For DNA elution, a volume of 30 µL elution buffer (Buffer EB, Qiagen (Hilden, Germany) was applied twice (making a total volume of 60 µL).

DNA purity was checked with the NanoDrop spectrophotometer (Thermo Fisher Scientific). For determination of the DNA concentration after DNA extraction, the Qubit 2.0 fluorometer in combination with the Qubit dsDNA BR Assay Kit (Thermo Fisher Scientific) was used according to the manufacturer’s instructions. Afterwards, the DNA concentration was adjusted to 5 ng/µL with 1× Tris-EDTA buffer (10 mM Tris-HCl, 1 mM EDTA, pH 8.0; Sigma-Aldrich, St. Louis, MO, USA).

### 2.4. Library Preparation and WGS

A volume of 55 µL DNA isolate was transferred to a microTUBE AFA Fiber Screw-Cap (Covaris, Woburn, MA, USA) for ultrasonification using Covaris M220 focused ultrasonicator (settings: peak incident power: 75 W, duty factor: 10%, cycles per burst: 200, treatment time: 60 s) to yield fragments with a mean fragment length of about 400 bp. Libraries were prepared with a DNA input of 250 ng using NEBNext Ultra II DNA Library Prep Kit for Illumina and NEBNext Multiplex Oligos for Illumina primer sets 1 and 2 (New England Biolabs (NEB), Ipswich, MA, USA), according to the manufacturer’s instructions. AMPure XP Beads (Beckman Coulter, Brea, CA, USA) were used for size selection and clean-up steps. Library concentrations were determined by Qubit 2.0 fluorometer in combination with the Qubit dsDNA HS Assay Kit (Thermo Fisher Scientific). Concentrations were adjusted with 1× Tris-EDTA buffer (10 mM Tris-HCl, 1 mM EDTA, pH 8.0; Sigma-Aldrich) to the range required for determination of the fragment size distribution checked by capillary electrophoresis (Fragment Analyzer; Agilent Technologies, Santa Clara, CA, USA) using the HS NGS Fragment Kit (Agilent Technologies).

Based on the fluorimetrically measured library concentrations and the average fragment size, the libraries were normalized manually to 2 nM with 1× Tris-EDTA buffer (10 mM Tris-HCl, 1 mM EDTA, pH 8.0; Sigma-Aldrich). Sequencing was performed on the Illumina MiSeq with MiSeq Reagent Kit v2 (500 cycles, Illumina, San Diego, CA, USA) with 2× 250 bp read length aiming for a coverage of about 70-fold. The run quality was evaluated by use of the Illumina Sequencing Analysis (SAV) software v 2.4.7.

### 2.5. In silico Typing

In silico serogrouping, MLST, and cgMLST was performed based on raw reads using the Ridom SeqSphere+ software v 7.0.2 (Ridom, Münster, Germany) [29] with default settings for quality checks and quality trimming, de novo assembly (SKESA v 2.3.0; [30]), and target scan procedure. Alleles were called for loci of three different schemes integrated in the task templates: (i) “*L. monocytogenes* 5-plex PCR Serogroup” v 1.0 comprising five loci [10], (ii) “*L. monocytogenes* MLST” v 1.0 comprising seven loci [11], and (iii) “*L. monocytogenes* cgMLST” v 2.1 comprising 1701 loci [12].

cgMLST allele distance matrices and minimum spanning trees were generated by pairwise ignoring missing values.

### 2.6. De novo Assembly and Quality Control

For the analysis of the pangenome and further characterization, assemblies generated with the AQUAMIS pipeline v 1.2.0 [31] were used. The pipeline comprises i. a. trimming of raw reads, de novo assembly, quality control of reads and assemblies, species identification, and a contamination check.

### 2.7. Pangenome Analysis

To annotate the assemblies generated with AQUAMIS, Prokka v 1.14.6 [32] was used. Based on these annotations, a pangenome was generated for each set using PIRATE v 1.0.3 [33], which allowed the identification of differences in the genome-wide gene presence/absence pattern between the isolates. The software was run on features annotated as CDS with 50, 60, 70, 80, 90, 95, and 98 amino acid % identity thresholds. All sequences belonging to the internal control LMO-C were analyzed by use of the PIRATE pipeline with paralog identification switched off.

### 2.8. Variant Calling

The snippySnake pipeline [34] based on snippy v 4.4.3 [35] was used for variant calling based on reads processed and trimmed by AQUAMIS. SNPs were called with and without filtering of SNPs in recombinant sites. The analysis was performed separately for groups of isolates of the same CT. Regarding the fish set, SNPs were called separately for isolates of the MLST sequence types (STs) ST2 and ST121. The optimal reference for each CT or ST was identified by the reffinder module integrated in the snippySnake pipeline. For the comparison of CT1248 sequences of set D or the LMO-C sequences, Lm strain 12-05460 (NZ_CP063381) was selected as reference. The used references for each set are listed in Table 1.

### 2.9. Genetic Traits

The assemblies of all 92 isolates were screened for a set of 295 genes in BIGSdb-*Lm* using the Gene Presence analysis tool with BLASTN algorithm as described by Moura et al. (2016) [14]. The set comprised genes of several schemes provided by the analysis tool, in particular, genes of the schemes *Virulence* [14], *Antibiotic Resistance* [14], *Metal and Disinfectants Resistance* [14], *Stress Islands* [36,37], *Listeria Genomic Islands* [38,39,40,41], *Rhamnose Operon* [42], and *sigB Operon* [43] as well as some single genes additionally analyzed in the study of Camargo et al. (2019) [44]. All analyzed genes are listed in Table S1. Allele IDs for *inlA* were determined and screened for allele IDs tagged as alleles with internal stop codons by using BIGSdb-*Lm*. For visualization of the gene presence/absence patterns (profiles), iTOL v 6 [45] was used.

### 2.10. Prophage Analyses

Putative prophages were identified by submitting all 92 assemblies generated with AQUAMIS to the PHASTER server through the URL API [46,47]. Phage profiling was performed based on the prophage name scored as the most common for each identified prophage region. In the cases where more than one top-hit prophage was identified for the same genome region, the same combinations of top-hit phages were merged and considered as distinct name categories for profiling. The same phage profiles were attributed to isolates having the same number of the same name categories with the same completeness classification.

## 3. Results

### 3.1. Reproducibility of WGS

To confirm the reproducibility of genome sequences generated with the described workflow, the Lm isolate LMO-C was included in 16 sequencing runs from cultivation to sequencing. cgMLST resulted in the same CT with zero allelic differences between all sequences of this isolate. Furthermore, no SNPs were detected for LMO-C repetitions by using the reference CP063381 [48], which belongs to the corresponding CT. Analysis of the pangenome showed no differences between nine LMO-C sequences. The residual seven sequences differed in the presence/absence of one to three genes, which were all present in the other nine sequences. Two of these genes were annotated as hypothetical proteins, whereas the third gene was assigned to an IS6 family transposase (IS1216E). The three genes were not located adjacent to each other in the isolates, in which all three genes were present. These differences should be considered as method variations when interpreting WGS data.

### 3.2. Genetic Diversity of Lm Food Isolates

**Figure 1:**
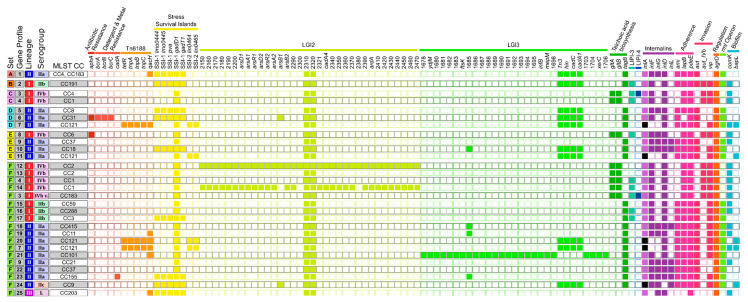
The 25 gene presence/absence profiles assigned to the 92 isolates according to the analyzed sets. The respective lineages, serogroups, and MLST CCs are also indicated. Full squares indicate detection, and empty squares no detection of the respective gene. Black squares depict *inlA* alleles with internal stop codons. Abbreviations: SSI, stress survival island; LGI, *Listeria* genomic island; LIPI, *Listeria* pathogenicity island.

In this study, 92 meat- and fish-associated Lm isolates were analyzed in distinct sets to determine the genetic diversity of spatially linked isolates and among isolates of the same matrix. Differences between isolates obtained from the same sample or processing plant were examined in order to assess the applicability of recommended clustering thresholds (Section 3.2.1 and Section 3.2.2, respectively). The genetic diversity between fish-associated isolates was determined to investigate potential persistence or transmission within the production chain (Section 3.2.3). Overall, the isolates analyzed in this study were assigned to 6 serogroups, 21 CCs, 21 STs, 41 CTs, 25 gene profiles, and 37 prophage profiles. Table 2, Table 3 and Table 4 show types and profile IDs for each isolate. Prophage profiles can be found in Table S3. The gene presence/absence profiles based on screening for 295 genes are presented in Figure 1 for genes found in at least one isolate. Genes absent or present in all isolates (Table S1) are not shown except for the genes LGI-2_LMOSA2310, LGI-2_LMOSA2320, and *gadD1*, as they are part of listeria genomic island (LGI) 2 and stress survival island (SSI) 1, respectively. 

#### 3.2.1. Genetic Diversity at Sample Level

The genetic diversity at sample level was determined by identifying differences between isolates that were obtained from the same sample. The respective isolates included all isolates of the sets A and B as well as individual isolates of the sets C and F (Table 2).

Set A

**Figure 2:**
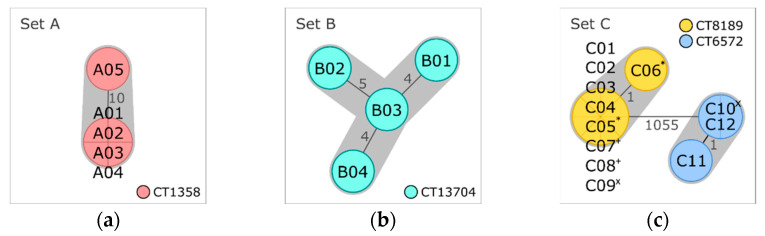
Minimum spanning trees based on core genome multilocus sequence typing (cgMLST) of (**a**) set A isolates obtained from the same meat sample, (**b**) set B isolates obtained from another meat sample, and (**c**) set C isolates obtained from processing plant 1. Isolates are represented in circles, which are colored according to their cgMLST type (CT). The number of allelic distances are shown next to the lines connecting the respective isolates. Clusters with allele difference ≤ 10 are shaded in gray. Superscripts mark set C isolates that were obtained from the same sample (* sample 7, ^+^ sample 8, and ^×^ sample 9).

All five isolates combined in set A were obtained from the same meat sample (Table 1). Typing results and gene profiles were congruent for all five isolates (Table 2). Four isolates were nearly indistinguishable, differing only in the gene content by zero to two pangenome genes (Tables S3–S5). The fifth isolate (A05) showed ten AD in pairwise comparison with the other isolates of the set (Figure 2a). SNP and pangenome analysis confirmed genetic divergence of the fifth isolate by detection of 802/24 SNPs (without/with filtering of recombinant sites) and 51 to 53 differences in gene presence/absence of the pangenome, respectively. A05 exhibited a phage profile other than the residual set A isolates (Table 2 and Table S2), indicating genome differences due to prophages. Inclusion of the prophage screening results could confirm that most pangenome differences and SNPs accumulated in genome regions identified as phage DNA. As no intermediate isolates were determined by the applied analysis methods, separation of A05 from the residual set A isolates might indicate co-occurrence of populations at different niches of the production chain, which have recently evolved from one ancestor cell, and cross contamination of the meat sample with representatives of both populations.

Set B

The four isolates of set B were obtained from another meat sample with no link to the sample of set A (Table 1). As for the latter, all set B isolates were assigned to the same lineage, serogroup, CC, ST, CT, and the same gene profile (Table 2). They differed in four to seven alleles (Figure 2b) and four to twenty SNPs (four to ten SNPs with filtering of recombinant sites) (Tables S6 and S7). Pangenome analysis separated isolate B04 from the residual isolates of set B by identifying 64 to 65 differences in gene presence/absence (Table S8). Of these genes, 63 to 64 were missing in B04 but present in the other three isolates. Comparison of the phage profiles revealed that B04 (phage profile PP02) lacks a genome region with the highest gene hit count for phage LP-101, which was present in all other set B isolates (PP01) (Table S2). The absence of this region was responsible for 63 of the 64 to 65 pangenomic differences, which were identified between B04 and the residual isolates of this set. The genetic divergence of set B isolates is not only represented by differences due to this single mobile element but also from independent SNPs in the core and the accessory genome, which could indicate persistence in the production chain over a longer period.

Set C isolates obtained from the same samples

Two isolates each were obtained from three of the nine samples belonging to meat-processing plant 1 (set C, Table 1). Figure 2c depicts the cgMLST minimum spanning tree of all set C isolates, which were examined as a whole in Section 3.2.2. Distance matrices are provided in Tables S9–S12. The pairwise comparison of the two isolates obtained from sample 7, C05 and C06, revealed a distance of one allele, two SNPs, and no difference in presence/absence of pangenome genes (Table 2). No difference could be determined between the two isolates of sample 8 (C07 and C08). In contrast, the isolates of sample 9, C09 and C10, differed by more than thousand alleles and the presence/absence of 144 genes of the pangenome, revealing the occurrence of two very distinct Lm subtypes within the same sample.

Set F isolates obtained from the same sample

The two isolates obtained from the same pangasius catfish sample, F10 and F11 (Table 1), differed from each other by 13 core genome alleles, 25 SNPs, and 0 pangenome genes (Table 2). They were assigned to different CTs but exhibited the same phage and gene profiles. The number of differences identified by cgMLST and SNP calling suggests long-term persistence and transmission to the fish sample at one site of the production chain or cross-contamination at different sites where populations of one recent ancestor have evolved independently.

#### 3.2.2. Genetic Diversity at Plant Level

The genetic diversity was also determined for Lm isolates that belonged to three meat-processing plants as described below. An overview of the typing and screening results as well as the number of differences between the isolates of the respective plants is provided in Table 3.

Processing Plant 1 (Set C)

The in silico analyses separated the twelve isolates of plant 1 into two distinct clusters, which differed from each other by more than a thousand alleles (Figure 2c) and the presence/absence of 144 to 146 pangenome genes (Tables S9 and S12). The nine isolates composing cluster 1 were assigned to lineage I, serogroup IVb, CC4, ST4, CT8189, and gene profile 3 (Table 3). The residual three isolates were attributed to the second cluster and grouped into lineage I, serogroup IVb, CC1, ST1, CT6572, and gene profile 4.

Within each cluster, the isolates were nearly identical (≤1 AD, ≤2 differences in the pangenome gene content, and ≤ 3 SNPs) (Table 3 and Tables S9–S12), supporting a close relatedness between these isolates. Despite the separation into two different CTs, all set C isolates exhibited the same phage profile (Table 3 and Table S2). The assigned gene profiles (3 and 4) differed by the *Listeria* pathogenicity island 4 (LIPI-4), which is involved in neural and placental tropisms [15] and was found in all CT8189 isolates but not in CT6572 isolates (Figure 1). The co-occurrence of the two CTs was also determined at sample level (see Section 3.2.1, set C). The small number of differences detected between the isolates of each CT does not indicate a long period of persistence or high mutation pressure in the processing environment.

Processing Plant 2 (Set D)

**Figure 3:**
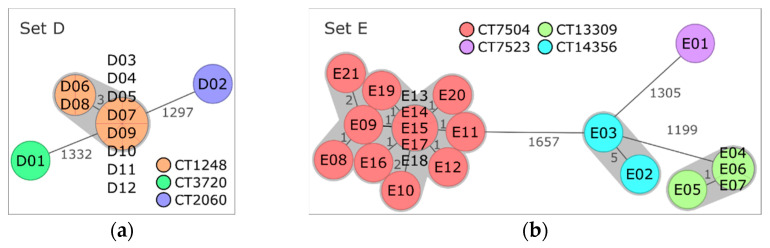
Minimum spanning trees based on core genome multilocus sequence typing (cgMLST) of (**a**) set D isolates obtained from processing plant 2 and (**b**) set E isolates from processing plant 3. Isolates are represented in circles, which are colored according to their cgMLST type (CT). The number of allelic distances are shown next to the lines connecting the respective isolates. Clusters with allele difference ≤ 10 are shaded in gray.

Ten out of twelve isolates originating from plant 2 were classified as lineage II, serogroup IIa, CC8, ST8, CT1248, and gene profile 5 (Table 3, Figure 3a). Pairwise comparison via cgMLST and pangenome analysis of CT1248 isolates resulted in a maximum AD of three alleles and zero to thirty-three differences in presence/absence of pangenome genes, respectively (Tables S13 and S14). Using a reference sequence belonging to the same CT for SNP calling (without filtering of recombinant sites) resulted in the separation of the 10 CT1248 isolates into two main subclusters, which differed from each other by 796 to 891 SNPs (Table S15). After removal of regions with elevated SNP counts, only few SNPs remained (Table 3). The vast majority of pangenome differences and removed SNPs were located within a genome region identified as prophage DNA. *Listeria* phages LP-101 and LP-HM00113468 (both scored as intact and with the same number of gene hits) or only intact LP-101 were predicted as the most common phage names for this region for all isolates (Table S2). Consequently, most genome differences could be attributed to modifications within a prophage region highly similar to LP-101 and LP-HM00113468.

cgMLST and pangenome analysis clearly separated the two residual set D isolates, D01 and D02, from each other and from CT1248 isolates (Tables S13 and S14). Thus, three distinct CTs were found on the samples of processing plant B.

According to the CTs, the isolates of set D were assigned to three different gene profiles (Table 3). All isolates of set D harbored genetic features potentially supporting their survival within food-processing plants, such as stress survival islands (SSIs) and/or detergent-resistance genes (Figure 1). A premature stop codon (PMSC) in *inlA*, which is associated with attenuated virulence [49], was detected in isolate D01. No obvious genetic factor could be identified as the reason for the predominance of CT1248 isolates in plant 2. Only *inlG* was found exclusively in the CT1248 isolates. This gene is suspected to contribute to the survival of Lm in the environment [50].

Processing Plant 3 (Set E)

The 21 isolates of plant 3 were grouped into three different clusters and one single isolate (Figure 3b). Fourteen isolates were assigned to lineage I, serogroup IVb, CC6, ST6, and CT7504, with AD ranging from zero to five and SNP distances ranging from zero to eight (Table 3, Tables S16 and S17). Pangenome analysis separated the isolates of CT7504 into two subclusters and one discrete isolate (E08) (Table S18). Most of the pangenome differences could be attributed to hypothetical *Listeria* spp. or phage-related genes. Separation of E08 in pangenome analysis due to phage DNA was also indicated by the assignment of a different phage profile to E08 (Table 3) and was mainly caused by the presence of intact phage B054 in all CT7504 isolates except E08 (Table S3).

The second most frequent CT of set E was CT13309 (lineage II, serogroup IIa, CC37, ST37) with four isolates. cgMLST, SNP, and pangenome analyses indicate a close relationship between these isolates (Table 3, Tables S16, S18 and S19). Similarly, the two isolates of the third cluster belonging to lineage II, serogroup IIa, CC18, ST18, and CT14356 showed only few differences in the core and the pangenome and a small number of SNPs (Table 3). The remaining isolate, E01, was assigned to lineage II, serogroup IIa, CC121, ST121, and CT7523. Comparison of the isolates of the four different CTs resulted in 1199 to 1666 AD and 308 to 628 differences in the pangenome gene presence/absence between the different CTs.

Noteworthy is the distribution of isolates over CTs and over years. The four isolates of plant C collected in 2018 were assigned to CT7523 (n = 1), CT14356 (n = 2), and CT7504 (n = 1). The two isolates collected in 2019 were classified as CT13309 and CT7504, respectively. All 15 isolates collected in 2020 were attributed either to CT13309 (n = 3) or CT7504 (n = 12). In summary, four different CTs were found for plant 3 over a three-year period, and only CT7504 was found in all three years.

Comparison of the gene profiles of the four CTs (Figure 1) revealed no apparent factor conferring advantages in the food-production environment specific for CT7504. Potentially, *gltA* and *gltB*, in set E only present in CT7504 isolates, represent candidate persistence genes. These genes are essential for the expression of teichoic acid-associated surface antigens in serotype 4b isolates [51], and *gltB* was shown to be involved in biofilm formation, adherence to glass surfaces, and tolerance against oxidative stress [52].

#### 3.2.3. Genetic Diversity of Fish Isolates (Set F)

**Figure 4:**
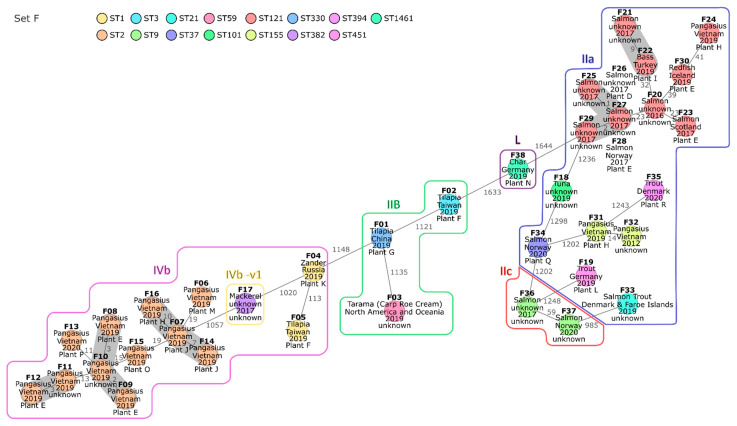
Minimum spanning tree of 38 isolates obtained from fish-associated samples based on core genome multilocus sequence typing (cgMLST). Isolates are represented in circles, which are colored according to their multilocus sequence type (ST). The number of allelic distances are shown next to the lines connecting the respective isolates. Clusters with allele difference ≤ 10 are shaded in gray. Isolates of the same serogroup are framed, and the respective serogroup is indicated next to the surrounding line. The isolates were annotated with the respective trade name, the country of origin, the year of sampling, and the processing plant inferred from the European Union (EU) approval number (when available).

Set F consisted of 38 isolates originating from 37 fish and fish products (35 fish samples, 1 roe cream, and 1 tuna wrap) (Table 1). cgMLST analysis classified all 38 isolates into 15 STs and 30 CTs (Table 4). A minimum spanning tree based on the cgMLST AD is shown in Figure 4. Strikingly, all ST2 isolates were obtained from fish traded as Vietnamese pangasius catfish (*Pangasianodon hypophthalmus*). Similarly, most of the isolates assigned to ST121 were obtained from salmon. The isolates of ST2 and ST121 were analyzed in more detail in order to investigate potential relationships within these STs.

The eleven pangasius catfish isolates of ST2 differed from each other by 2 to 29 alleles of the core genome (Table 4 and Table S20). The number of SNPs detected between the ST2 fish isolates was low to moderate, ranging from 3 to 54 (Table S21). Nine of the ST2 fish isolates differed from each other by the presence/absence of maximum one gene (Table S22). The residual two ST2 fish isolates, F16 and F15, varied from the latter in the presence/absence of up to 36 and 159 genes, respectively. The presence of LGI2, which harbors arsenic- and cadmium-resistance genes [41] in all ST2 fish isolates (gene profile 12) except F15 and F16 (both gene profile 13), was responsible for 34 differences in gene presence/absence (Figure 1). The residual pangenome differences between F15 and the other ST2 fish isolates could be mainly attributed to genome regions assigned to an incomplete *Corynebacterium* and an intact vB LmoS 188 prophage (Table S2). In summary, the differences in pangenome gene presence/absence within the ST2 fish cluster were largely induced by a small number of mobile elements.

The two isolates obtained from the same pangasius catfish sample were also assigned to ST2 and differed from each other by 13 alleles. The ADs between isolates of different processing plants were partly lower. For instance, the isolate F13 of plant P differed from the isolates F08 and F09 of plant E by 12 and 11 alleles, respectively. The occurrence of isolates with a relatively close relatedness in distinct plants indicates contaminations within the primary production.

The ST121 fish cluster was more diverse than the ST2 fish cluster. The AD between the eleven ST121 fish isolates ranged from zero to fifty-six alleles (Table 4 and Table S20). cgMLST and SNP analysis indicated a close relationship between the isolates F25 to F29, all obtained from salmon samples in 2017 (Tables S20 and S23). The respective samples were processed in plant D, plant E, or in an unknown plant. The pangenome gene presence/absence ranged from zero to three between four of the five isolates, confirming a close relatedness for these isolates (Table S22). Almost all pangenome genes missing in the fifth isolate, F29, were located within two different genome regions in F25 to F28 and attributed to phage genes. As plant D and E are subsidiaries of the same company, they possibly process fish from the same primary production, or one supplies the other for further processing of commodities. This could explain the occurrence of very closely related isolates in fish samples obtained from these plants.

Noteworthy is the detection of a ST382 isolate (F17) obtained from a mackerel sample in 2017. Since ST382 was linked to several outbreaks, it was previously classified as epidemic clone [13]. As known for ST382 isolates [53], F17 exhibits the hypervirulence factor LIPI-4 (gene profile 3, Figure 1).

## 4. Discussion

Interpretation of WGS data during outbreak investigations needs to consider the genetic diversity of Lm isolates within processing plants and along potential transmission routes [24]. In the present study, Lm isolates obtained from food-associated samples collected in the scope of official monitoring in Bavaria were subjected to WGS and analyzed with in silico typing approaches as well as screening for genetic traits and prophages. The aims of the study were (i) to examine the reproducibility of genome sequences generated with the described workflow and to identify potential genome variations, (ii) to determine the genetic diversity at sample and at plant level and to assess the recommended clustering thresholds, and (iii) to investigate the genomic diversity of fish-associated samples potentially indicating persistence or transmission within the production chain.

### 4.1. High Reproducibility of Genome Sequences

The sequences of an internal control were compared via cgMLST, pangenome analysis, and SNP calling in order to examine whether repetitive preparation of the same isolate results in reproducible genome sequences. While cgMLST and SNP analyses revealed no differences between LMO-C sequences, a variation in the presence/absence of three genes was detected by pangenome analysis. This variation might be explained by assembly errors leading to the loss of single genes. It is known that misassemblies of the draft genome can affect the number of inferred genes [54,55].

One of the three genes was annotated as an insertion sequence (IS1216E). Integration or excision of this small autonomous transposable element upon cultivation at different time points may be the reason for the respective gene presence/absence.

Differences determined by repetitive analysis of the same isolate are regarded as method variations and need to be considered in cluster interpretations. Keeping in mind that a small number of genes are potentially not represented in the assembly, the established workflow yielded highly reproducible genome sequences. Additionally, reproducibility and consistency of sequence quality was also confirmed within an interlaboratoy study, which compared genome sequences generated by different institutions using their in-house protocols for library preparation and sequencing [56].

### 4.2. High Range of Diverstiy between Spatially Linked Isolates

Reference distance thresholds are used in order to assess whether genome comparisons indicate a potential relatedness between clinical and food isolates. Strict thresholds are impractical, as the genetic diversity varies within different populations and transmission at several steps of the food-processing chain needs to be considered [24]. However, detailed knowledge of *Listeria* evolution in processing plants is rare [57]. Fundamental to the data interpretation within an outbreak investigation is the awareness of the approximate number of differences within populations originating from a suspicious processing plant. Diversity between the respective food isolates and related clinical isolates can be slightly higher.

In this study, Lm isolates belonging to the same samples or the same processing plants were analyzed in distinct sets in order determine the genetic diversity at sample and at plant level, respectively. Isolates obtained from the same processing plants differed by 0 to 1666 core genome alleles and were partly assigned to distinct CTs, MLST clones, serogroups, and lineages. More than one CT was found within all investigated processing plants. These results reflect the potential coexistence of distinct subtypes in processing plants already reported in previous studies [17,18,19,20,21,22]. Some of these subtypes are known to be able to persist in processing plants and were linked to several outbreaks: CC8, for instance, persisted in various plants that processed different kinds of food matrices [58,59,60] and was linked to outbreaks, i.e., in Canada [61], Germany [48], and to a multicountry outbreak in Europe [62]. Other subtypes, which were assigned to isolates analyzed within this study, such as CC1, CC2, CC4, and CC6, were also designated as epidemic clones as they have been associated with more than one outbreak [13]. The analysis of representatives of these subtypes within this study may help to increase the understanding of the potential extent of the genetic diversity between spatially linked isolates attributed to outbreak-associated subtypes.

Genetic diversity was also found at sample level. For one sample, the two obtained isolates (C09 and C10) diverged clearly by differing in more than thousand alleles. ADs between the residual isolates obtained from the same samples ranged between zero and 13. The isolation of different subtypes from a single sample were reported previously [23,63,64,65]. Analysis of a small number of samples and isolates obtained from food or the respective processing environment may be insufficient to identify representatives of all populations and, therefore, could lead to missed matches between infection sources and clinical isolates during outbreak investigations [23].

Comprehensive sampling enables not only the probability to identify potentially outbreak-related subtypes [23] but also the determination of variations thereof (e.g., due to phage insertions or SNPs) [66]. In this study, genomic differences between isolates, which were obtained from the same sample or processing plant and had likely evolved from one recent ancestor cell, ranged between zero and 13 ADs and zero to 25 SNPs (with filtering of recombinant sites). Thus, the suggested thresholds for cluster identification (≤7 to 12 ADs for 1701-loci cgMLST [12,67] or up to about 20 SNPs [24,68]) were mostly appropriate. In case the determined diversity within a given environment is slightly higher, the threshold might be carefully adjusted. As genomic differences might accumulate, i.a., within the course of disease, it should also be considered that distances between food-associated and clinical isolates might be even larger [25].

Pangenome analyses are usually not suitable for clustering of outbreak-related isolates, as the clusters can correspond more to phage types than to common ancestry [69]. However, comparison of gene content by pangenome analysis enables the determination of further differences in the accessory genome. After pre-clustering by cgMLST, we applied SNP calling (without filtering of recombinant regions) as well as pangenome analyses in order to get an in-depth view of potential genome variations. Isolates assigned to the same CT and originating from the same processing plant differed from each other by zero to 891 SNPs and in the presence/absence of 0 to 80 pangenome genes. Most of these differences accumulated at specific genome regions. Comparison of these regions with the results of the screenings for prophages and genetic traits revealed that the accumulated differences could be mainly attributed to a few mobile genetic elements (MGEs), such as phages and genomic islands. Similarly, Harrand et al. (2020) [57] observed that evolution of Lm in processing plants is mainly driven by gain or loss of prophages and to a lesser extent by independent SNPs. MGEs can be used as genetic markers strengthening the attribution of isolates to an outbreak or giving evidence that isolates are potentially unrelated when exhibiting distinct MGE profiles [70,71]. The determination of different MGE profiles for isolates obtained from the same processing plant, as determined for prophages in this study, might increase the probability of source identification in outbreak analyses.

### 4.3. Predominant Isolation of Specific Subtypes from Pangasius Catfish and Salmon Samples

The 38 fish-associated isolates analyzed in this study exhibited a large range of diversity by differing in 0 to 1666 core genome alleles and in the presence/absence of zero to 687 pangenome genes.

Most isolates obtained from pangasius catfish were assigned to ST2. None of the residual isolates from other fish species in this study were attributed to this ST. The genetic diversity determined for ST2 fish isolates indicated that some of them were very closely related, whereas the accumulation of genetic differences has led to divergence between others.

All pangasius catfish samples originated from aquacultures in the Mekong Delta of Vietnam. The EU is one of the main customers of Vietnamese pangasius catfish [72], and major efforts have been made in Vietnam to fulfill the quality and food safety standards stipulated by the EU and further importing countries [73].

The performance of food safety management systems implemented in distinct plants processing pangasius catfish was assessed in different studies: Lm could not be determined in a large-scale plant, in which the pathogen had sporadically occurred before [74]. The examination of the whole production process of a small-scale plant revealed the presence of Lm in one final product [73]. However, high prevalence of Lm was determined in another plant where the pathogen was detected i.a. in raw and processed fish as well as on contact surfaces [75].

Whether contaminations of pangasius source from raw fish or from the processing environment (or both) is a controversial question [73]. Lm is able to survive several weeks in samples of pond water and soil [76] and was determined in flowing surface waters [77]. It was also detected in water of pangasius catfish ponds in Vietnam [78].

The water of ponds, in which pangasius catfish is usually reared, is mostly derived from and drained into the Mekong River [79]. These results could potentially indicate persistence of ST2 isolates in the ponds used for aquaculture of pangasius catfish. Draining of contaminated pond water into the Mekong River and use of the river water for pond filling might have been a way of transmission to further fish farms. Processing of contaminated fish may have led to the dissemination and persistence in the respective plants. Ponds and processing plants present ecological niches where the ST2 isolates could have evolved independently.

Two isolates obtained from the same pangasius catfish sample (F10 and F11) differed in 13 alleles, 25 SNPs, and 0 pangenome genes. This might be a result of long-time persistence within aquaculture ponds or processing plants. It is also conceivable that the occurrence of subtype variants within a single sample is due to cross-contaminations at different steps of pangasius catfish rearing and processing.

Screening for genetic traits revealed that ST2 isolates might be well-adapted to the Mekong Delta region. Most of the ST2 fish isolates in this study harbored LGI2 linked with arsenic and cadmium resistance [41,80]. Arsenic was found in high concentrations in groundwater of the Mekong Delta [81]. Furthermore, high concentrations of arsenic and cadmium in the mud of some shrimp-farming ponds in coastal regions of Vietnam were reported [82].

As we detected LGI2 also in ST2 isolates from meat (isolates not included in this study), presence of this genomic island is likely not due to the adaption to high concentrations of these pollutants. Nevertheless, carriage of LGI2 might be beneficial for the survival of ST2 isolates in this region. Other STs not harboring LGI2 were only detected for 3 of 14 isolates from pangasius catfish. ST2 belongs to the hypervirulent MLST clone CC2. This presents a certain hazard, as introduction of pangasius catfish contaminated with Lm into domestic kitchens or environments of processing plants may lead to the exposure of humans due to cross contaminations or insufficient heating [83].

Similar to the predominant assignment of pangasius catfish isolates to ST2, most of the isolates obtained from salmon were grouped into ST121. The attribution to ST2 or ST121 was not species-specific, as individual isolates of pangasius catfish and salmon were classified to other STs. Besides salmon, three isolates from other fish species (bass, redfish, and pangasius catfish) were classified into ST121. The ST121 fish isolates exhibited a larger range of diversity than the ST2 pangasius catfish isolates analyzed in this study.

Only few differences were found between ST121 salmon isolates obtained from subsidiaries of the same company indicating a common source of contamination due to supply with raw commodities of the same origin or transmission by consecutive processing of the same product. The residual ST121 fish isolates were more diverse so that common descent of one recent ancestor was rather unlikely. ST121 belongs to the food-associated clone CC121, which is regarded as hypovirulent due to PMSCs in *inlA* [15]. Nevertheless, this subtype sometimes caused human infections [15,84]. Since ready-to-eat salmon products have not necessarily passed a thorough heating process, contaminations with ST121 should not be underestimated.

ST121 was already determined in processing plants of salmon [85,86] and turned out as persistent subtype in several food-processing environments [87,88,89,90]. ST121 genomes are highly conserved—independent of source, time, and space—differing mainly in prophage content and regions [84]. As shown for ST8, transmission of persistent clones between various processing plants is possible, e.g., due to contaminated raw materials processed in more than one plant or due to the exchange of processing equipment [91].

Persistence and transmission of conserved subtypes within the rearing and processing chain may lead to outbreaks, which cannot be linked to one specific plant by WGS methods. In such situations, the combination of analysis approaches applied in this study reaches its limits, as very similar subtype variants may occur within distinct plants. This underlines the need for supporting epidemiological data [25].

It has to be noted that the results might be biased through supply chains as well as sampling and thus may be not representative for salmon and pangasius catfish products. Therefore, further investigations are required to determine the diversity of ST121 and ST2 within the rearing and processing chains. These examinations could also provide the information regarding whether the detected ST121 and ST2 variants source from feedstocks or processing plants.

Noteworthy, one of the fish isolates (F17) was assigned to ST382. This ST was designated as epidemic clone since it was attributed to more than one outbreak [13]. Carriage of the hypervirulence factor LIPI-4 is common for ST382, and contamination of food presents a public health concern [53]. The respective isolate was obtained from a mackerel sample in 2017. As for some of the other fish samples, tracing back the supply chain was hampered since information about processing plants and country of origin was lacking. Labeling of the mackerel sample with “Sgombro”, the Italian name for mackerel, might indicate the Mediterranean Sea as origin. ST382 was previously considered as an emerging clone particularly associated with produce in the United States [13], and CC183 (comprising ST382) was determined as the second most common clone in public access surface waters in an agricultural region of the Central California Coast [92]. ST382 has to the best of our knowledge not been reported for food isolates in Europe to date.

## 5. Conclusions

In this study, we analyzed the genetic diversity within sets of meat- or fish-associated Lm isolates. Initially, we determined that the described workflow yields highly reproducible genome sequences, which may differ in the presence of a small number of single genes.

Analysis of the genetic diversity of spatially linked isolates revealed not only the co-occurrence of distinct subtypes but also variations between closely related isolates, which could be mainly attributed to MGEs. cgMLST has turned out beneficial for pre-clustering of closely related isolates when isolates slightly exceeding the suggested clustering threshold of ≤12 ADs were also considered. Analyses of the pangenome enable the determination of differences in accessory genome regions, which can be consulted to strengthen or to object to the evidence that isolates are linked with each other.

Furthermore, analysis of fish-associated isolates resulted in a predominant assignment of pangasius catfish and salmon to specific STs. Genomic congruence and genetic traits indicated persistence and selection advantage of ST2 within the production chain of pangasius catfish. Genome comparisons portended dissemination or transmission of ST121 within the salmon processing chain of one company, but the analyses did not support the conclusion that the residual ST121 isolates from salmon have recently diverged from one ancestor cell.

Finally, the described combination of WGS-based methods can enhance the understanding of Lm persistence and transmission within food-production chains and supports the interpretation of WGS data in outbreak investigations due to increased discriminatory power. A prerequisite is comprehensive sampling, which allows the identification of different subtypes and variations thereof.

## Figures and Tables

**Table 1:** Overview of all isolates analyzed in this study and their classification into different sets. Isolates obtained from the same samples are written in bold.

Set	Isolation Source	Year of Sampling	No. of Isolates	Isolate IDs	RefSeq Accession No. Reference for SNP Calling
**Sets with different subcultures of the same sample each**					
Set A	Smoked sausage	2019	5	**A01 to A05**	NZ_HG813247.1 NZ_HG813248.1
Set B	Blood sausage	2020	4	**B01 to B04**	NC_021824.1
**Sets of isolates originating from the same processing plant**					
Set C	Meat and meat products and environment of processing plant A	2020	12	C01 to C12 **Two isolates per sample:** **Sample 7: C05 and C06** **Sample 8: C07 and C08** **Sample 9: C09 and C10** One isolate per sample: C01 to C04, C11, C12	CT8189: NC_018642.1 CT6572: NC_019556.1
Set D	Meat and meat products and vegetarian products of processing plant B	2016	12	D01 to D12	CT1248: NZ_CP063381.1
Set E	Meat and meat products of processing plant C		Total: 21	E01 to E21	CT7504: NZ_CP010346.1 CT13309: NZ_CP007198.1 CT14356: NZ_CP020830.1
		2018	4	E01 to E03, E09	
		2019	2	E04, E10	
		2020	15	E05 to E08, E11 to E21	
**Fish Set**					
Set F	Fish		Total: 38	F01 to F38	ST2: NZ_CP013288.1 ST121: NZ_HG813249.1 NZ_HG813250.1
		2012	1	F32	
		2016	1	F20	
		2017	9	F17, F21, F23, F25 to F29, F36	
		2019	23	**Two isolates per sample** **Sample 54: F10 and F11** One isolate per sample: F01 to F09, F12, F14 to F16, F18, F19, F22, F24, F30, F31, F33, F38	
		2020	4	F13, F34, F35, F37	

**Table 2:** Set isolates obtained from the same sample with indication of typing and screening results as well as the number of differences detected between the isolates by different analysis methods. The samples combined within each set had no known link to samples of one of the other sets.

Set	Sample ID	Isolate ID	Lineage	Serogroup	CC ^1^	ST ^2^	CT ^3^	Gene Profile	Phage Profile (PP)	cgMLST Allelic Distance	Pangenome Gene Differences	SNPs ^4^ (without Filtering)	SNPs ^4^ (with Filtering)
Set A	1	A01	II	IIa	CC8	8	1358	1	PP33	0–10	0–53	0–802	0–24
		A02											
		A03											
		A04											
		A05							PP34				
Set B	2	B01	I	IIb	CC191	191	13,704	2	PP01	4–7	1–65	4–20	4–10
		B02											
		B03											
		B04							PP02				
Set C *	7	C05	I	IVb	CC4	4	8189	3	PP05	1	0	2	2
		C06											
	8	C07								0	0	0	0
		C08											
	9	C09								1.057	1	N/A	N/A
		C10			CC1	1	6572	4					
Set F *	54	F10	I	IVb	CC2	2	8488	12	PP08	13	0	25	25
		F11					8489						

* Only set isolates that were obtained from the same sample(s); ^1^ CC, clonal complex based on multilocus sequence typing (MLST); ^2^ ST, sequence type based on MLST; ^3^ CT, complex type based on core genome MLST (cgMLST); ^4^ SNPs, single-nucleotide polymorphisms.

**Table 3:** Set isolates obtained from the same processing plants with indication of typing and screening results as well as the number of differences detected between the isolates by different analysis methods.

Set	Sample ID	Isolate ID	Lineage	Serogroup	CC	ST	CT	Gene Profile (GP)	Phage Profile (PP)	cgMLST Allelic Distance	Pangenome Gene Differences	SNPs (without Filtering)	SNPs (with Filtering)
Set C (Plant 1)	3	C01	I	IVb	CC4	4	8189	3	PP05	0–1	0–2	0–3	0–3
	4	C02											
	5	C03											
	6	C04											
	7	C05											
		C06											
	8	C07											
		C08											
	9	C09											
		C10			CC1	1	6572	4		0–1	0	1–2	1–2
	10	C11											
	11	C12											
Set D (Plant 2)	12	D01	II	IIa	CC121	121	3720	7	PP14	N/A	N/A	N/A	N/A
	13	D02			CC31	325	2060	6	PP27	N/A	N/A	N/A	N/A
	14	D03			CC8	8	1248	5	PP31	0–3	0–33	0–891	0–9
	15	D04											
	16	D05											
	17	D06							PP32				
	18	D07											
	19	D08											
	20	D09											
	21	D10											
	22	D11											
	23	D12											
Set E (Plant 3)	24	E01	II	IIa	CC121	121	7523	11	PP22	N/A	N/A	N/A	N/A
	25	E02			CC18	18	14,356	10	PP24	5	3	11	N/A
	26	E03							PP25				
	27	E04			CC37	37	13,309	9	PP29	0–1	0–1	1–4	0–4
	28	E05											
	29	E06											
	30	E07											
	31	E08	I	IVb	CC6	6	7504	8	PP02	0–5	0–80	0–8	0–8
	32	E09							PP10				
	33	E10											
	34	E11											
	35	E12											
	36	E13											
	37	E14											
	38	E15											
	39	E16											
	40	E17											
	41	E18											
	42	E19											
	43	E20											
	44	E21											

**Table 4:** Set F isolates obtained from fish-associated samples with indication of typing and screening results as well as the number of differences detected between the isolates by different analysis methods.

Set	Sample ID	Isolate ID	Lineage	Serogroup	CC	ST	CT	Gene Profile	Phage Profile (PP)	Allelic Distance	Pangenome Gene Differences	SNPs (without Filtering)	SNPs (with Filtering)
Set F	45	F01 ^1^	I	IIb	CC288	330	8487	16	PP03	NA	NA	NA	NA
	46	F02 ^1^			CC3	3	4792	17	PP04	NA	NA	NA	NA
	47	F03 ^2^			CC59	59	9054	15	PP02	NA	NA	NA	NA
	48	F04 ^3^		IVb	CC1	1	8948	4	PP06	NA	NA	NA	NA
	49	F05 ^1^					13,330	14	PP07	NA	NA	NA	NA
	50	F06 ^4^			CC2	2	4240	12	PP08	2–29	0–159	3–54	3–54
	51	F07 ^4^					5312						
	52	F08 ^4^					8488						
	53	F09 ^4^											
	54	F10 ^4^											
		F11 ^4^					8489						
	55	F12 ^4^											
	56	F13 ^4^					14,148						
	57	F14 ^4^					14,725						
	58	F15 ^4^					6639	13					
	59	F16 ^4^					13,038		PP09				
	60	F17 ^5^		IVb v.	CC183	382	2944	3	PP02	NA	NA	NA	NA
	61	F18 ^6^	II	IIa	CC101	101	8424	21	PP11	NA	NA	NA	NA
	62	F19 ^7^			CC11	451	13,697	19	PP12	NA	NA	NA	NA
	63	F20 ^8^			CC121	121	2278	20	PP13	0–56	0–267	2–115	2–115
	64	F21 ^8^					4295		PP15				
	65	F22 ^9^							PP16				
	66	F23 ^8^					4507		PP17				
	67	F24 ^4^					9100		PP18				
	68	F25 ^8^					5554						
	69	F26 ^8^											
	70	F27 ^8^											
	71	F28 ^8^							PP20				
	72	F29 ^8^						7	PP19				
	73	F30 ^10^					6097		PP21				
	74	F31 ^4^			CC155	155	2842	23	PP23	NA	NA	NA	NA
	75	F32 ^4^					8952			NA	NA	NA	NA
	76	F33 ^11^			CC21	21	8946	9	PP26	NA	NA	NA	NA
	77	F34 ^8^			CC37	37	7559	22	PP28	NA	NA	NA	NA
	78	F35 ^7^			CC415	394	14,488	18	PP30	NA	NA	NA	NA
	79	F36 ^8^		IIc	CC9	9	92	24	PP35	NA	NA	NA	NA
	80	F37 ^8^					1690		PP36	NA	NA	NA	NA
	81	F38 ^12^	III	L	CC203	1461	2761	25	PP37	NA	NA	NA	NA

^1^ Tilapia; ^2^ Tarama; ^3^ Zander; ^4^ Pangasius; ^5^ Mackerel; ^6^ Tuna (wrap); ^7^ Trout; ^8^ Salmon; ^9^ Bass; ^10^ Redfish; ^11^ Salmon Trout; ^12^ Char.

## Data Availability

Short-read sequencing data for all isolates analyzed in this study can be found online deposited in the NCBI database under the BioProject number PRJNA893902.

## Supplementary Materials

The following supporting information can be downloaded at: https://www.mdpi.com/article/10.3390/microorganisms10112120/s1, Table S1: Set of genes screened for presence within this study; Table S2: Prophage Profiles of each isolate sorted by lineage, serogroup, CC, ST, and CT; File S1: Tables S3–S23: Distance matrices obtained by cgMLST, pangenome analysis, and SNP calling for all sets.
