# Supplementary material for: Whole-Genome Sequence Comparisons of Listeria monocytogenes Isolated from Meat and Fish Reveal High Inter- and Intra-Sample Diversity
Source: Microorganisms. 2022 Oct 26;10(11):2120. doi: 10.3390/microorganisms10112120 (PMC9698462; doi:10.3390/microorganisms10112120)
Supplement: Supplementary file 1 [file microorganisms-10-02120-s001.zip › File_S1.pdf]

Supplementary Material

1 Genetic Traits

Table S1. Set of genes screened for presence within this study.

(see File Table_S1.xlsx)

2 Prophage Profiling

Table S2. Prophage Profiles of each isolate sorted by lineage, serogroup, CC, ST and CT.

(see File Table_S2.xlsx)

3 Distance Matrices

The following tables depict cgMLST, SNP and pangenomic distances arising from the pairwise comparison of the isolates of the different sets. The coloring of distance numbers with graduated color scales serves for illustration and is based on fixed values. No or only few distances are colored in green, moderate distances (15, 100 and 10 distances for cgMLST, SNP calling (without filtering of genome regions with elevated SNP counts) and pangenome analyses, respectively) are shown in yellow, whereas high distance numbers (100, 500 and 100 distances for cgMLST, SNP calling (without filtering) and pangenome analyses, respectively) are indicated in red.

3.1 Set A

Table S3. cgMLST allele distance matrix of set A isolates.

	A04	A03	A01	A02	A05
A04	0	0	0	0	10
A03	0	0	0	0	10
A01	0	0	0	0	10
A02	0	0	0	0	10
A05	10	10	10	10	0

Table S4. SNP distance matrix of set A isolates. Numbers separated by a slash indicate differences determined by SNP calling without/with filtering of recombinant sites.

	A01	A02	A03	A04	A05	Reference
A01	0	0	0	0	802/24	1117/37
A02	0	0	0	0	802/24	1117/37
A03	0	0	0	0	802/24	1117/37
A04	0	0	0	0	802/24	1117/37
A05	802/24	802/24	802/24	802/24	0	1475/45
Reference	1117/37	1117/37	1117/37	1117/37	1475/45	0

Table S5. Distance matrix with pangenome gene presence/absence differences between set A isolates.

	A04	A03	A01	A02	A05
A04	0	2	2	0	51
A03	2	0	0	2	53
A01	2	0	0	2	53
A02	0	2	2	0	51
A05	51	53	53	51	0

3.1.1.1 Set B

Table S6. cgMLST allele distance matrix of set B isolates.

	B01	B02	B03	B04
B01	0	7	4	6
B02	7	0	5	5
B03	4	5	0	4
B04	6	5	4	0

Table S7. SNP distance matrix of set B isolates. Numbers separated by a slash indicate differences determined by SNP calling without/with filtering of recombinant sites.

	B01	B02	B03	B04	Reference
B01	0	12/10	4/4	18/8	10754/6879
B02	12/10	0	12/10	20/8	10758/6883
B03	4/4	12/10	0	18/8	10754/6879
B04	18/8	20/8	18/8	0	10757/6881
Reference	10754/6879	10758/6883	10754/6879	10757/6881	0

Table S8. Distance matrix with pangenome gene presence/absence differences between set B isolates.

	B01	B02	B03	B04
B01	0	1	2	65
B02	1	0	1	64
B03	2	1	0	65
B04	65	64	65	0

3.1.1.2 Set C

Table S9. cgMLST allele distance matrix of set C isolates.
Identical coloring of the isolate IDs marks isolates extracted from the same samples.

	C10	C11	C12	C05	C01	C07	C08	C09	C02	C06	C03	C04
C10	0	1	0	1057	1057	1055	1056	1057	1057	1058	1057	1057
C11	1	0	1	1058	1058	1056	1057	1058	1058	1059	1058	1058
C12	0	1	0	1057	1057	1055	1056	1057	1057	1058	1057	1057
C05	1057	1058	1057	0	0	0	0	0	0	1	0	0
C01	1057	1058	1057	0	0	0	0	0	0	1	0	0
C07	1055	1056	1055	0	0	0	0	0	0	1	0	0
C08	1056	1057	1056	0	0	0	0	0	0	1	0	0
C09	1057	1058	1057	0	0	0	0	0	0	1	0	0
C02	1057	1058	1057	0	0	0	0	0	0	1	0	0
C06	1058	1059	1058	1	1	1	1	1	1	0	1	1
C03	1057	1058	1057	0	0	0	0	0	0	1	0	0
C04	1057	1058	1057	0	0	0	0	0	0	1	0	0

Table S10. SNP distance matrix of CT8189 isolates of set C. SNP counts were congruent for SNP calling with and without filtering of recombinant sites. Identical coloring of the isolate IDs marks isolates extracted from the same samples.

**CT8189**	C01	C02	C03	C04	C05	C06	C07	C08	C09	Reference
C01	0	2	2	2	2	2	3	3	2	111
C02	2	0	0	0	0	2	1	1	0	109
C03	2	0	0	0	0	2	1	1	0	109
C04	2	0	0	0	0	2	1	1	0	109
C05	2	0	0	0	0	2	1	1	0	109
C06	2	2	2	2	2	0	3	3	2	111
C07	3	1	1	1	1	3	0	0	1	110
C08	3	1	1	1	1	3	0	0	1	110
C09	2	0	0	0	0	2	1	1	0	109
Reference	111	109	109	109	109	111	110	110	109	0

Table S11. SNP distance matrix of CT6572 isolates of set C. SNP counts were congruent for SNP calling with and without filtering of recombinant sites.

**CT6572**	C10	C11	C12	Reference
C10	0	1	1	89
C11	1	0	2	90
C12	1	2	0	90
Reference	89	90	90	0

Table S12. Distance matrix with pangenome gene presence/absence differences between set C isolates.

	C10	C11	C12	C05	C01	C07	C08	C09	C02	C06	C03	C04
C10	0	0	0	144	146	145	145	144	145	144	145	144
C11	0	0	0	144	146	145	145	144	145	144	145	144
C12	0	0	0	144	146	145	145	144	145	144	145	144
C05	144	144	144	0	2	1	1	2	1	0	1	2
C01	146	146	146	2	0	1	1	2	1	2	1	2
C07	145	145	145	1	1	0	0	1	0	1	0	1
C08	145	145	145	1	1	0	0	1	0	1	0	1
C09	144	144	144	2	2	1	1	0	1	2	1	0
C02	145	145	145	1	1	0	0	1	0	1	0	1
C06	144	144	144	0	2	1	1	2	1	0	1	2
C03	145	145	145	1	1	0	0	1	0	1	0	1
C04	144	144	144	2	2	1	1	0	1	2	1	0

3.1.1.3 Set D

Table S13. cgMLST allele distance matrix of set D isolates.

	D03	D07	D04	D05	D09	D10	D11	D12	D06	D08	D02	D01
D03	0	0	0	0	0	0	0	0	3	3	1297	1332
D07	0	0	0	0	0	0	0	0	3	3	1297	1332
D04	0	0	0	0	0	0	0	0	3	3	1297	1332
D05	0	0	0	0	0	0	0	0	3	3	1297	1332
D09	0	0	0	0	0	0	0	0	3	3	1297	1332
D10	0	0	0	0	0	0	0	0	3	3	1297	1332
D11	0	0	0	0	0	0	0	0	3	3	1297	1332
D12	0	0	0	0	0	0	0	0	3	3	1297	1332
D06	3	3	3	3	3	3	3	3	0	0	1297	1332
D08	3	3	3	3	3	3	3	3	0	0	1297	1332
D02	1297	1297	1297	1297	1297	1297	1297	1297	1297	1297	0	1358
D01	1332	1332	1332	1332	1332	1332	1332	1332	1332	1332	1358	0

Table S14. Distance matrix with pangenome gene presence/absence differences between set D isolates.

	D07	D09	D12	D05	D03	D04	D10	D11	D06	D08	D02	D01
D07	0	1	0	18	28	27	31	31	31	32	513	424
D09	1	0	1	19	27	28	32	32	32	33	514	425
D12	0	1	0	18	28	27	31	31	31	32	513	424
D05	18	19	18	0	12	11	13	13	15	14	521	430
D03	28	27	28	12	0	1	7	7	5	6	519	426
D04	27	28	27	11	1	0	6	6	4	5	518	425
D10	31	32	31	13	7	6	0	0	2	1	522	423
D11	31	32	31	13	7	6	0	0	2	1	522	423
D06	31	32	31	15	5	4	2	2	0	1	522	423
D08	32	33	32	14	6	5	1	1	1	0	523	422
D02	513	514	513	521	519	518	522	522	522	523	0	467
D01	424	425	424	430	426	425	423	423	423	422	467	0

Table S15. SNP distance matrix of CT1248 isolates of set D. Numbers separated by a slash indicate differences determined by SNP calling without/with filtering of recombinant sites.

**CT1248**	D07	D09	D12	D05	D03	D04	D10	D11	D06	D08	Reference
D07	0	2/1	2/1	85/1	89/1	89/1	881/0	881/0	885/4	889/8	888/7
D09	2/1	0	0	83/0	87/0	89/2	883/1	883/1	887/5	891/9	890/8
D12	2/1	0	0	83/0	87/0	89/2	883/1	883/1	887/5	891/9	890/8
D05	85/1	83/0	83/0	0	104/0	106/2	800/1	800/1	804/5	808/9	807/8
D03	89/1	87/0	87/0	104/0	0	2	796/1	796/1	800/5	804/9	803/8
D04	89/1	89/2	89/2	106/2	2	0	796/1	796/1	800/5	804/9	803/8
D10	881/0	883/1	883/1	800/1	796/1	796/1	0	0	4	8	7
D11	881/0	883/1	883/1	800/1	796/1	796/1	0	0	4	8	7
D06	885/4	887/5	887/5	804/5	800/5	800/5	4	4	0	4	5
D08	889/8	891/9	891/9	808/9	804/9	804/9	8	8	4	0	9
Reference	888/7	890/8	890/8	807/8	803/8	803/8	7	7	5	9	0

3.1.1.4 Set E

Table S16. cgMLST allele distance matrix of set E isolates.

	E01	E02	E03	E04	E05	E06	E07	E09	E10	E11	E12	E08	E13	E14	E15	E16	E17	E18	E19	E20	E21
E01	0	1307	1305	1321	1321	1321	1321	1663	1663	1662	1663	1663	1663	1663	1663	1663	1663	1663	1663	1662	1663
E02	1307	0	5	1201	1202	1201	1201	1658	1658	1657	1658	1658	1658	1658	1658	1658	1658	1658	1658	1657	1658
E03	1305	5	0	1199	1200	1199	1199	1658	1658	1657	1658	1658	1658	1658	1658	1658	1658	1658	1658	1657	1658
E04	1321	1201	1199	0	1	0	0	1666	1666	1665	1666	1666	1666	1666	1666	1666	1666	1666	1666	1665	1666
E05	1321	1202	1200	1	0	1	1	1666	1666	1665	1666	1666	1666	1666	1666	1666	1666	1666	1666	1665	1666
E06	1321	1201	1199	0	1	0	0	1666	1666	1665	1666	1666	1666	1666	1666	1666	1666	1666	1666	1665	1666
E07	1321	1201	1199	0	1	0	0	1666	1666	1665	1666	1666	1666	1666	1666	1666	1666	1666	1666	1665	1666
E09	1663	1658	1658	1666	1666	1666	1666	0	3	2	2	1	1	1	1	2	1	1	2	2	2
E10	1663	1658	1658	1666	1666	1666	1666	3	0	3	3	4	2	2	2	3	2	2	3	3	5
E11	1662	1657	1657	1665	1665	1665	1665	2	3	0	2	3	1	1	1	2	1	1	2	2	4
E12	1663	1658	1658	1666	1666	1666	1666	2	3	2	0	3	1	1	1	2	1	1	2	2	4
E08	1663	1658	1658	1666	1666	1666	1666	1	4	3	3	0	2	2	2	3	2	2	3	3	3
E13	1663	1658	1658	1666	1666	1666	1666	1	2	1	1	2	0	0	0	1	0	0	1	1	3
E14	1663	1658	1658	1666	1666	1666	1666	1	2	1	1	2	0	0	0	1	0	0	1	1	3
E15	1663	1658	1658	1666	1666	1666	1666	1	2	1	1	2	0	0	0	1	0	0	1	1	3
E16	1663	1658	1658	1666	1666	1666	1666	2	3	2	2	3	1	1	1	0	1	1	2	2	4
E17	1663	1658	1658	1666	1666	1666	1666	1	2	1	1	2	0	0	0	1	0	0	1	1	3
E18	1663	1658	1658	1666	1666	1666	1666	1	2	1	1	2	0	0	0	1	0	0	1	1	3
E19	1663	1658	1658	1666	1666	1666	1666	2	3	2	2	3	1	1	1	2	1	1	0	2	4
E20	1662	1657	1657	1665	1665	1665	1665	2	3	2	2	3	1	1	1	2	1	1	2	0	4
E21	1663	1658	1658	1666	1666	1666	1666	2	5	4	4	3	3	3	3	4	3	3	4	4	0

Table S17. SNP distance matrix of CT7504 isolates of set E. Numbers separated by a slash indicate differences determined by SNP calling without/with filtering of recombinant sites.

**CT7504**	E08	E09	E10	E11	E12	E13	E14	E15	E16	E17	E18	E19	E20	E21	Reference
E08	0	3/2	5	5	3	3	2	2	4	3	4	3	6	4	71/69
E09	3/2	0	6/5	6/5	4/3	4/3	3/2	3/2	5/4	4/3	5/4	4/3	7/6	5/4	70/69
E10	5	6/5	0	6	4	4	3	3	5	4	5	4	7	7	74/72
E11	5	6/5	6	0	4	4	3	3	5	4	5	4	7	7	74/72
E12	3	4/3	4	4	0	2	1	1	3	2	3	2	5	5	72/70
E13	3	4/3	4	4	2	0	1	1	3	2	3	2	5	5	72/70
E14	2	3/2	3	3	1	1	0	0	2	1	2	1	4	4	71/69
E15	2	3/2	3	3	1	1	0	0	2	1	2	1	4	4	71/69
E16	4	5/4	5	5	3	3	2	2	0	3	2	3	6	6	73/71
E17	3	4/3	4	4	2	2	1	1	3	0	3	2	5	5	72/70
E18	4	5/4	5	5	3	3	2	2	2	3	0	3	6	6	73/71
E19	3	4/3	4	4	2	2	1	1	3	2	3	0	5	5	72/70
E20	6	7/6	7	7	5	5	4	4	6	5	6	5	0	8	75/73
E21	4	5/4	7	7	5	5	4	4	6	5	6	5	8	0	73/71
Reference	71/69	70/69	74/72	74/72	72/70	72/70	71/69	71/69	73/71	72/70	73/71	72/70	75/73	73/71	0

Table S18. Distance matrix with pangenome gene presence/absence differences between set E isolates.

CT	7523	14356		13309				7504														
ID	E01	E02	E03	E04	E05	E06	E07	E08	E09	E11	E20	E10	E14	E15	E18	E13	E16	E19	E21	E12	E17
E01	0	421	418	434	434	435	434	628	584	586	585	577	577	577	577	576	576	576	576	578	577
E02	421	0	3	311	311	310	311	479	543	545	544	550	550	550	550	549	549	549	549	551	550
E03	418	3	0	308	308	309	308	476	540	542	541	547	547	547	547	546	546	546	546	548	547
E04	434	311	308	0	0	1	0	362	432	434	433	437	437	437	437	436	436	436	436	436	435
E05	434	311	308	0	0	1	0	362	432	434	433	437	437	437	437	436	436	436	436	436	435
E06	435	310	309	1	1	0	1	363	433	435	434	438	438	438	438	437	437	437	437	437	436
E07	434	311	308	0	0	1	0	362	432	434	433	437	437	437	437	436	436	436	436	436	435
E08	628	479	476	362	362	363	362	0	78	80	79	77	77	77	77	76	76	76	76	78	77
E09	584	543	540	432	432	433	432	78	0	2	1	19	19	19	19	18	18	18	18	18	17
E11	586	545	542	434	434	435	434	80	2	0	3	19	19	19	19	20	20	20	20	18	19
E20	585	544	541	433	433	434	433	79	1	3	0	20	20	20	20	19	19	19	19	19	18
E10	577	550	547	437	437	438	437	77	19	19	20	0	0	0	0	1	1	1	1	1	2
E14	577	550	547	437	437	438	437	77	19	19	20	0	0	0	0	1	1	1	1	1	2
E15	577	550	547	437	437	438	437	77	19	19	20	0	0	0	0	1	1	1	1	1	2
E18	577	550	547	437	437	438	437	77	19	19	20	0	0	0	0	1	1	1	1	1	2
E13	576	549	546	436	436	437	436	76	18	20	19	1	1	1	1	0	0	0	0	2	1
E16	576	549	546	436	436	437	436	76	18	20	19	1	1	1	1	0	0	0	0	2	1
E19	576	549	546	436	436	437	436	76	18	20	19	1	1	1	1	0	0	0	0	2	1
E21	576	549	546	436	436	437	436	76	18	20	19	1	1	1	1	0	0	0	0	2	1
E12	578	551	548	436	436	437	436	78	18	18	19	1	1	1	1	2	2	2	2	0	1
E17	577	550	547	435	435	436	435	77	17	19	18	2	2	2	2	1	1	1	1	1	0

Table S19. SNP distance matrix of CT13309 isolates of set E. Numbers separated by a slash indicate differences determined by SNP calling without/with filtering of recombinant sites.

**CT13309**	E04	E05	E06	E07	Reference
E04	0	2	2	1/0	458/91
E05	2	0	4	3/2	460/93
E06	2	4	0	3/2	460/93
E07	1/0	3/2	3/2	0	459/91
Reference	458/91	460/93	460/93	459/91	0

3.1.1.5 Set F

Table S20. cgMLST allele distance matrix of set F isolates. Isolates of ST2 and ST121 are marked in green and red, respectively. The isolates F10 and F11 are highlighted as they were extracted from the same sample.

								ST2															ST121															
	F03	F05	F01	F38	F17	F02	F04	F06	F16	F07	F14	F15	F12	F11	F09	F10	F08	F13	F31	F18	F32	F35	F30	F24	F21	F22	F23	F20	F25	F26	F28	F27	F29	F19	F34	F36	F33	F37
F03	0	1166	1135	1641	1162	1146	1161	1183	1182	1181	1184	1184	1182	1182	1182	1182	1183	1181	1658	1654	1657	1660	1663	1663	1663	1663	1664	1664	1664	1664	1664	1664	1663	1663	1664	1663	1662	1663
F05	1166	0	1157	1637	1039	1157	113	1098	1100	1099	1100	1097	1095	1096	1095	1095	1096	1095	1657	1654	1656	1658	1659	1659	1659	1659	1660	1660	1660	1660	1660	1660	1659	1661	1662	1661	1660	1661
F01	1135	1157	0	1636	1171	1121	1148	1201	1201	1201	1202	1202	1200	1201	1201	1202	1203	1201	1653	1650	1652	1654	1657	1657	1657	1657	1658	1658	1658	1658	1658	1658	1657	1658	1659	1658	1657	1658
F38	1641	1637	1636	0	1639	1633	1637	1639	1640	1640	1640	1640	1640	1641	1639	1641	1641	1641	1650	1644	1649	1655	1644	1644	1644	1644	1645	1645	1645	1645	1645	1645	1644	1652	1650	1658	1654	1658
F17	1162	1039	1171	1639	0	1152	1020	1058	1058	1057	1058	1059	1060	1061	1059	1059	1060	1059	1659	1654	1658	1659	1660	1660	1660	1660	1661	1661	1661	1661	1661	1661	1660	1663	1663	1661	1660	1661
F02	1146	1157	1121	1633	1152	0	1151	1190	1187	1188	1190	1189	1186	1187	1189	1188	1188	1187	1655	1649	1654	1656	1657	1657	1657	1657	1658	1658	1658	1658	1658	1658	1657	1659	1660	1659	1658	1659
F04	1161	113	1148	1637	1020	1151	0	1085	1087	1086	1087	1084	1083	1084	1082	1082	1083	1082	1657	1654	1656	1658	1660	1660	1660	1660	1661	1661	1661	1661	1661	1661	1660	1661	1662	1661	1660	1661
F06	1183	1098	1201	1639	1058	1190	1085	0	21	19	21	25	29	28	25	25	26	26	1659	1656	1658	1660	1661	1661	1661	1661	1662	1662	1662	1662	1662	1662	1661	1664	1664	1663	1662	1663
F16	1182	1100	1201	1640	1058	1187	1087	21	0	10	12	21	26	25	22	22	23	23	1660	1657	1659	1661	1662	1662	1662	1662	1663	1663	1663	1663	1663	1663	1662	1665	1665	1664	1663	1664
F07	1181	1099	1201	1640	1057	1188	1086	19	10	0	7	19	24	23	20	20	21	21	1660	1657	1659	1661	1662	1662	1662	1662	1663	1663	1663	1663	1663	1663	1662	1665	1665	1664	1663	1664
F14	1184	1100	1202	1640	1058	1190	1087	21	12	7	0	22	27	26	23	23	24	24	1660	1657	1659	1661	1662	1662	1662	1662	1663	1663	1663	1663	1663	1663	1662	1665	1665	1664	1663	1664
F15	1184	1097	1202	1640	1059	1189	1084	25	21	19	22	0	20	19	15	15	16	17	1660	1657	1659	1661	1662	1662	1662	1662	1663	1663	1663	1663	1663	1663	1662	1665	1665	1664	1663	1664
F12	1182	1095	1200	1640	1060	1186	1083	29	26	24	27	20	0	3	14	14	15	15	1660	1657	1659	1661	1662	1662	1662	1662	1663	1663	1663	1663	1663	1663	1662	1665	1665	1664	1663	1664
F11	1182	1096	1201	1641	1061	1187	1084	28	25	23	26	19	3	0	13	13	14	14	1661	1658	1660	1662	1663	1663	1663	1663	1664	1664	1664	1664	1664	1664	1663	1666	1666	1665	1664	1665
F09	1182	1095	1201	1639	1059	1189	1082	25	22	20	23	15	14	13	0	2	3	11	1659	1656	1658	1660	1661	1661	1661	1661	1662	1662	1662	1662	1662	1662	1661	1664	1664	1663	1662	1663
F10	1182	1095	1202	1641	1059	1188	1082	25	22	20	23	15	14	13	2	0	3	11	1661	1658	1660	1662	1663	1663	1663	1663	1664	1664	1664	1664	1664	1664	1663	1666	1666	1665	1664	1665
F08	1183	1096	1203	1641	1060	1188	1083	26	23	21	24	16	15	14	3	3	0	12	1661	1658	1660	1662	1663	1663	1663	1663	1664	1664	1664	1664	1664	1664	1663	1666	1666	1665	1664	1665
F13	1181	1095	1201	1641	1059	1187	1082	26	23	21	24	17	15	14	11	11	12	0	1661	1658	1660	1662	1663	1663	1663	1663	1664	1664	1664	1664	1664	1664	1663	1666	1666	1665	1664	1665
F31	1658	1657	1653	1650	1659	1655	1657	1659	1660	1660	1660	1660	1660	1661	1659	1661	1661	1661	0	1303	14	1243	1355	1352	1353	1352	1354	1354	1353	1353	1353	1353	1353	1251	1202	1249	1271	1250
F18	1654	1654	1650	1644	1654	1649	1654	1656	1657	1657	1657	1657	1657	1658	1656	1658	1658	1658	1303	0	1303	1316	1240	1239	1239	1238	1236	1237	1237	1237	1237	1237	1236	1314	1298	1304	1315	1305
F32	1657	1656	1652	1649	1658	1654	1656	1658	1659	1659	1659	1659	1659	1660	1658	1660	1660	1660	14	1303	0	1246	1356	1353	1354	1353	1355	1355	1354	1354	1354	1354	1354	1251	1203	1249	1274	1250
F35	1660	1658	1654	1655	1659	1656	1658	1660	1661	1661	1661	1661	1661	1662	1660	1662	1662	1662	1243	1316	1246	0	1342	1344	1341	1340	1342	1341	1342	1342	1342	1342	1341	1262	1261	1266	1266	1267
F30	1663	1659	1657	1644	1660	1657	1660	1661	1662	1662	1662	1662	1662	1663	1661	1663	1663	1663	1355	1240	1356	1342	0	41	53	52	43	39	45	44	44	44	44	1341	1322	1328	1336	1332
F24	1663	1659	1657	1644	1660	1657	1660	1661	1662	1662	1662	1662	1662	1663	1661	1663	1663	1663	1352	1239	1353	1344	41	0	56	55	46	42	48	47	47	47	47	1340	1322	1327	1336	1331
F21	1663	1659	1657	1644	1660	1657	1660	1661	1662	1662	1662	1662	1662	1663	1661	1663	1663	1663	1353	1239	1354	1341	53	56	0	9	36	33	38	38	38	38	38	1338	1322	1326	1333	1330
F22	1663	1659	1657	1644	1660	1657	1660	1661	1662	1662	1662	1662	1662	1663	1661	1663	1663	1663	1352	1238	1353	1340	52	55	9	0	35	32	37	37	37	37	37	1338	1323	1326	1332	1330
F23	1664	1660	1658	1645	1661	1658	1661	1662	1663	1663	1663	1663	1663	1664	1662	1664	1664	1664	1354	1236	1355	1342	43	46	36	35	0	23	29	28	28	28	28	1339	1324	1327	1335	1331
F20	1664	1660	1658	1645	1661	1658	1661	1662	1663	1663	1663	1663	1663	1664	1662	1664	1664	1664	1354	1237	1355	1341	39	42	33	32	23	0	24	23	23	23	23	1338	1325	1327	1334	1331
F25	1664	1660	1658	1645	1661	1658	1661	1662	1663	1663	1663	1663	1663	1664	1662	1664	1664	1664	1353	1237	1354	1342	45	48	38	37	29	24	0	1	1	1	3	1340	1324	1329	1334	1332
F26	1664	1660	1658	1645	1661	1658	1661	1662	1663	1663	1663	1663	1663	1664	1662	1664	1664	1664	1353	1237	1354	1342	44	47	38	37	28	23	1	0	0	0	2	1340	1324	1328	1334	1331
F28	1664	1660	1658	1645	1661	1658	1661	1662	1663	1663	1663	1663	1663	1664	1662	1664	1664	1664	1353	1237	1354	1342	44	47	38	37	28	23	1	0	0	0	2	1340	1324	1328	1334	1331
F27	1664	1660	1658	1645	1661	1658	1661	1662	1663	1663	1663	1663	1663	1664	1662	1664	1664	1664	1353	1237	1354	1342	44	47	38	37	28	23	1	0	0	0	2	1340	1324	1328	1334	1331
F29	1663	1659	1657	1644	1660	1657	1660	1661	1662	1662	1662	1662	1662	1663	1661	1663	1663	1663	1353	1236	1354	1341	44	47	38	37	28	23	3	2	2	2	0	1339	1324	1327	1333	1330
F19	1663	1661	1658	1652	1663	1659	1661	1664	1665	1665	1665	1665	1665	1666	1664	1666	1666	1666	1251	1314	1251	1262	1341	1340	1338	1338	1339	1338	1340	1340	1340	1340	1339	0	1250	1248	1260	1250
F34	1664	1662	1659	1650	1663	1660	1662	1664	1665	1665	1665	1665	1665	1666	1664	1666	1666	1666	1202	1298	1203	1261	1322	1322	1322	1323	1324	1325	1324	1324	1324	1324	1324	1250	0	1202	1207	1204
F36	1663	1661	1658	1658	1661	1659	1661	1663	1664	1664	1664	1664	1664	1665	1663	1665	1665	1665	1249	1304	1249	1266	1328	1327	1326	1326	1327	1327	1329	1328	1328	1328	1327	1248	1202	0	986	59
F33	1662	1660	1657	1654	1660	1658	1660	1662	1663	1663	1663	1663	1663	1664	1662	1664	1664	1664	1271	1315	1274	1266	1336	1336	1333	1332	1335	1334	1334	1334	1334	1334	1333	1260	1207	986	0	985
F37	1663	1661	1658	1658	1661	1659	1661	1663	1664	1664	1664	1664	1664	1665	1663	1665	1665	1665	1250	1305	1250	1267	1332	1331	1330	1330	1331	1331	1332	1331	1331	1331	1330	1250	1204	59	985	0

Table S21. SNP distance matrix of ST2 isolates of set F. SNP counts were congruent for SNP calling with and without filtering of recombinant sites.

ST2	F09	F10	F08	F13	F11	F12	F16	F07	F14	F06	F15	Reference
F09	0	4	5	24	27	28	44	43	45	50	36	150
F10	4	0	3	22	25	26	42	41	43	48	34	148
F08	5	3	0	23	26	27	43	42	44	49	35	149
F13	24	22	23	0	27	28	44	43	45	50	36	150
F11	27	25	26	27	0	9	47	46	48	53	39	153
F12	28	26	27	28	9	0	48	47	49	54	40	154
F16	44	42	43	44	47	48	0	19	21	40	38	144
F07	43	41	42	43	46	47	19	0	12	39	37	143
F14	45	43	44	45	48	49	21	12	0	41	39	145
F06	50	48	49	50	53	54	40	39	41	0	44	150
F15	36	34	35	36	39	40	38	37	39	44	0	144
Reference	150	148	149	150	153	154	144	143	145	150	144	0

Table S22. Distance matrix with pangenome gene presence/absence differences between set F isolates.

	ST121																				ST2																	
	F20	F30	F22	F21	F23	F29	F28	F27	F25	F26	F24	F18	F33	F19	F35	F36	F37	F34	F31	F32	F15	F16	F07	F13	F09	F06	F08	F14	F12	F11	F10	F38	F02	F01	F04	F05	F17	F03
F20	0	163	147	144	142	94	100	100	98	99	99	461	298	309	345	366	407	339	301	301	524	452	485	486	486	486	486	486	486	486	486	449	471	464	508	600	457	407
F30	163	0	266	263	267	177	223	223	221	222	222	502	327	342	312	417	370	456	428	428	403	475	508	509	509	509	509	509	509	509	509	402	436	457	533	565	410	360
F22	147	266	0	49	71	129	115	115	113	114	114	432	377	334	388	395	428	328	332	332	625	547	580	581	581	581	581	581	581	581	581	550	492	509	567	649	558	510
F21	144	263	49	0	58	104	94	94	94	95	95	419	374	327	365	376	405	313	315	315	622	538	571	572	572	572	572	572	572	572	572	547	471	486	554	648	555	507
F23	142	267	71	58	0	106	60	62	62	63	63	405	366	299	369	384	391	307	313	313	626	536	569	570	570	570	570	570	570	570	570	551	465	486	558	652	559	509
F29	94	177	129	104	106	0	48	48	48	49	49	435	346	283	283	312	381	333	285	285	536	492	525	526	526	526	526	526	526	526	526	461	439	442	476	576	469	421
F28	100	223	115	94	60	48	0	2	2	3	3	423	332	277	325	346	361	315	279	279	582	496	529	530	530	530	530	530	530	530	530	507	427	442	518	610	515	465
F27	100	223	115	94	62	48	2	0	2	1	1	423	332	277	325	346	359	315	277	277	582	496	529	530	530	530	530	530	530	530	530	507	427	442	518	610	515	465
F25	98	221	113	94	62	48	2	2	0	1	1	421	330	275	323	344	361	315	279	279	580	496	529	530	530	530	530	530	530	530	530	505	427	442	518	610	513	463
F26	99	222	114	95	63	49	3	1	1	0	0	422	331	276	324	345	360	316	278	278	581	497	530	531	531	531	531	531	531	531	531	506	428	443	519	611	514	464
F24	99	222	114	95	63	49	3	1	1	0	0	422	331	276	324	345	360	316	278	278	581	497	530	531	531	531	531	531	531	531	531	506	428	443	519	611	514	464
F18	461	502	432	419	405	435	423	423	421	422	422	0	425	344	370	457	440	392	394	394	635	567	600	601	601	601	601	601	601	601	601	526	516	511	601	687	562	544
F33	298	327	377	374	366	346	332	332	330	331	331	425	0	269	281	330	307	335	319	319	474	398	429	430	430	430	430	430	430	430	430	409	423	408	482	586	389	339
F19	309	342	334	327	299	283	277	277	275	276	276	344	269	0	230	317	282	274	226	226	447	395	428	429	429	429	429	429	429	429	429	412	334	323	443	555	378	350
F35	345	312	388	365	369	283	325	325	323	324	324	370	281	230	0	287	288	314	296	296	435	427	460	459	459	459	459	459	459	459	459	360	374	383	439	541	348	312
F36	366	417	395	376	384	312	346	346	344	345	345	457	330	317	287	0	197	329	297	297	562	534	563	564	564	564	564	564	564	564	564	467	453	456	500	600	465	419
F37	407	370	428	405	391	381	361	359	361	360	360	440	307	282	288	197	0	352	330	330	499	501	530	531	531	531	531	531	531	531	531	476	308	389	547	569	476	428
F34	339	456	328	313	307	333	315	315	315	316	316	392	335	274	314	329	352	0	266	266	553	469	502	503	503	503	503	503	503	503	503	514	444	443	503	591	492	458
F31	301	428	332	315	313	285	279	277	279	278	278	394	319	226	296	297	330	266	0	0	533	465	498	497	497	497	497	497	497	497	497	466	374	387	469	567	456	416
F32	301	428	332	315	313	285	279	277	279	278	278	394	319	226	296	297	330	266	0	0	533	465	498	497	497	497	497	497	497	497	497	466	374	387	469	567	456	416
F15	524	403	625	622	626	536	582	582	580	581	581	635	474	447	435	562	499	553	533	533	0	126	159	158	158	158	158	158	158	158	158	357	363	350	284	318	205	281
F16	452	475	547	538	536	492	496	496	496	497	497	567	398	395	427	534	501	469	465	465	126	0	35	36	36	36	36	36	36	36	36	357	359	332	236	328	205	279
F07	485	508	580	571	569	525	529	529	529	530	530	600	429	428	460	563	530	502	498	498	159	35	0	1	1	1	1	1	1	1	1	390	392	365	269	295	236	312
F13	486	509	581	572	570	526	530	530	530	531	531	601	430	429	459	564	531	503	497	497	158	36	1	0	0	0	0	0	0	0	0	389	391	364	268	294	235	311
F09	486	509	581	572	570	526	530	530	530	531	531	601	430	429	459	564	531	503	497	497	158	36	1	0	0	0	0	0	0	0	0	389	391	364	268	294	235	311
F06	486	509	581	572	570	526	530	530	530	531	531	601	430	429	459	564	531	503	497	497	158	36	1	0	0	0	0	0	0	0	0	389	391	364	268	294	235	311
F08	486	509	581	572	570	526	530	530	530	531	531	601	430	429	459	564	531	503	497	497	158	36	1	0	0	0	0	0	0	0	0	389	391	364	268	294	235	311
F14	486	509	581	572	570	526	530	530	530	531	531	601	430	429	459	564	531	503	497	497	158	36	1	0	0	0	0	0	0	0	0	389	391	364	268	294	235	311
F12	486	509	581	572	570	526	530	530	530	531	531	601	430	429	459	564	531	503	497	497	158	36	1	0	0	0	0	0	0	0	0	389	391	364	268	294	235	311
F11	486	509	581	572	570	526	530	530	530	531	531	601	430	429	459	564	531	503	497	497	158	36	1	0	0	0	0	0	0	0	0	389	391	364	268	294	235	311
F10	486	509	581	572	570	526	530	530	530	531	531	601	430	429	459	564	531	503	497	497	158	36	1	0	0	0	0	0	0	0	0	389	391	364	268	294	235	311
F38	449	402	550	547	551	461	507	507	505	506	506	526	409	412	360	467	476	514	466	466	357	357	390	389	389	389	389	389	389	389	389	0	446	443	415	517	310	318
F02	471	436	492	471	465	439	427	427	427	428	428	516	423	334	374	453	308	444	374	374	363	359	392	391	391	391	391	391	391	391	391	446	0	217	401	421	350	296
F01	464	457	509	486	486	442	442	442	442	443	443	511	408	323	383	456	389	443	387	387	350	332	365	364	364	364	364	364	364	364	364	443	217	0	392	452	325	287
F04	508	533	567	554	558	476	518	518	518	519	519	601	482	443	439	500	547	503	469	469	284	236	269	268	268	268	268	268	268	268	268	415	401	392	0	180	251	307
F05	600	565	649	648	652	576	610	610	610	611	611	687	586	555	541	600	569	591	567	567	318	328	295	294	294	294	294	294	294	294	294	517	421	452	180	0	343	407
F17	457	410	558	555	559	469	515	515	513	514	514	562	389	378	348	465	476	492	456	456	205	205	236	235	235	235	235	235	235	235	235	310	350	325	251	343	0	202
F03	407	360	510	507	509	421	465	465	463	464	464	544	339	350	312	419	428	458	416	416	281	279	312	311	311	311	311	311	311	311	311	318	296	287	307	407	202	0

Table S23. SNP distance matrix of ST121 isolates of set F. SNP counts were congruent for SNP calling with and without filtering of recombinant sites.

**ST121**	F29	F26	F27	F25	F28	F30	F24	F21	F22	F23	F20	Reference
F29	0	9	9	10	10	74	96	71	77	58	52	54
F26	9	0	2	3	3	75	97	72	78	59	53	55
F27	9	2	0	3	3	75	97	72	78	59	53	55
F25	10	3	3	0	4	76	98	73	79	60	54	56
F28	10	3	3	4	0	76	98	73	79	60	54	56
F30	74	75	75	76	76	0	78	87	93	74	68	70
F24	96	97	97	98	98	78	0	109	115	96	90	92
F21	71	72	72	73	73	87	109	0	26	61	63	65
F22	77	78	78	79	79	93	115	26	0	67	69	71
F23	58	59	59	60	60	74	96	61	67	0	50	52
F20	52	53	53	54	54	68	90	63	69	50	0	44
Reference	54	55	55	56	56	70	92	65	71	52	44	0
